# Study of the Effects Induced by Ball Milling Treatment on Different Types of Hydrocolloids in a Corn Starch–Rice Flour System

**DOI:** 10.3390/foods9040517

**Published:** 2020-04-20

**Authors:** Luca Nuvoli, Paola Conte, Sebastiano Garroni, Valeria Farina, Antonio Piga, Costantino Fadda

**Affiliations:** 1 Department of Chemistry and Pharmacy, University of Sassari and INSTM, Via Vienna 2, 07100 Sassari, Italy; lunuvoli@uniss.it (L.N.); sgarroni@uniss.it (S.G.); valefari89@gmail.com (V.F.); 2 Department of Agriculture, University of Sassari, Viale Italia 39/A, 07100 Sassari, Italy; pigaa@uniss.it (A.P.); cfadda@uniss.it (C.F.)

**Keywords:** ball milling, hydrocolloids, starch–flour system, X-ray diffraction, pasting profile, viscoelastic properties

## Abstract

The effects of ball milling treatment on both the structure and properties of guar gum (GG), tara gum (TG), and methylcellulose (MC) were analyzed prior to assessing their potential interactions with starch components when they are used alone or in blends in a corn starch–rice flour system. X-ray diffraction profiles showed that the ball milling caused a reduction in the crystallin domain and, in turn, a diminished viscosity of the GG aqueous solutions. Despite an increase in its viscosity properties, effects on TG were minimal, while the milled MC exhibited reduced crystallinity, but similar viscosity. When both milled and un-milled hydrocolloids were individually added to the starch–flour system, the pasting properties of the resulting mixtures seemed to be affected by the type of hydrocolloid added rather than the structural changes induced by the treatment. All hydrocolloids increased the peak viscosity of the binary blends (especially pure GG), but only milled and un-milled MC showed values of setback and final viscosity similar to those of the individual starch. Ball milling seemed to be more effective when two combined hydrocolloids (milled GG and MC) were simultaneously used. No significant differences were observed in the viscoelastic properties of the blends, except for un-milled GG/starch, milled TG/starch, and milled MC/milled TG/starch gels.

## 1. Introduction

The use of starch and its ability to gelatinize make it a necessary ingredient in food systems [1]. Starch is a macroconstituent of many foods and it is composed of amylopectin and amylose. The organization of the crystalline lamellae within granules is due to the packing of amylopectin into crystallites and this phenomenon is influenced by amylose. The amylopectin structure and the role of amylose are important for the water swelling of starch granules, the thermal properties and gel formation (in water solution) [2]. However, when starch is subjected to processes, such as cooking, shear stress, and cooling, it tends to exhibit syneresis, retrogradation, and breakdown, suggesting that its use as a main ingredient in food may be difficult [3]. To overcome these issues, hydrophilic colloids are often used to modify the functionality of starch [4]. Hydrophilic colloids, commonly known as “hydrocolloids”, are substances consisting of long chains that have a high molecular weight and an affinity for water, which, in a water-based system, generate gels or highly viscous suspensions. Typically, hydrocolloids are polysaccharides, composed of single or multiple units of sugar, repeated over the entire length of the polymer. Their properties are then influenced by external units, such as sulfate groups, methyl ether groups or carboxyl groups, linked to the main chain [5,6]. The ability to form networks, once dispersed in a solution, and their biocompatibility, makes them excellent additives and/or substituents in food chemistry for the control of microstructure, texture, flavor, and shelf-life [7,8]. Among others, the naturally occurring polysaccharides, such as galactomannans, as well as the chemically synthesized cellulose derivatives, have been widely used in the food industry for their suitable functional properties. In particular, the cellulose derivative methylcellulose has many applications in food systems such as an emulsifier and texturing agent, as well as a thickener and gelling additive [9], whereas galactomannans, such as guar gum and tara gum, are well known to have thickening, binding, and stabilizing abilities [10]. These two galactomannans, however, despite a very similar molecular structure—composed of a β-(1–4)-d-mannan backbone with a single d-galactose branch linked α-(1–6)—differ from each other in the molar ratio of mannose to galactose (3:1 for tara and 2:1 for guar gum), which is responsible for the significant changes in their viscosity and solubility properties, as well as in their interactions with other polysaccharides [10]. The authors of [3] explored the interactions of rice starch with guar gam, hydroxypropylmethylcellulose (HPMC), and xanthan gum to improve the pasting, viscoelastic, and swelling properties of starch–hydrocolloid mixtures. To determine the network stability, the effects of repeated heating–cooling cycles were also analyzed. They found that the tested rice starch–hydrocolloid mixtures (8%, *w*/*w*) exhibited different pasting, viscoelastic, and swelling properties depending on the type of hydrocolloid added, as well as the concentration levels (0.2%–0.8% *w*/*w*) used. The authors of [11], when investigating the swelling and pasting properties of non-waxy rice starch–hydrocolloid mixtures using both commercial and laboratory-generated hydrocolloids, observed a strong dependence between the hydrocolloid concentration and swelling power of the rice starch–hydrocolloid blends. They found that the swelling behavior was depressed at low concentrations of hydrocolloids (0%–0.05%), but it increased linearly with increasing hydrocolloid concentrations (0.05%–0.1%). Other hydrocolloids (e.g., alginate, k-carrageenan, xanthan) were investigated by [12] with two different techniques. Recently, it was demonstrated that the use of mechanical treatments, such as ball milling, can alter the functionality of hydrocolloids, influencing, in turn, their interactions with the starch or its constituents [13,14,15]. Changes in hydrocolloid functionality are probably due to the changes induced by the milling treatment on their structural conformation, thermal properties, particle size distributions, and rheological properties, enhancing the amorphization of the system at the expense of the crystalline structure. The authors of [16] investigated the effects of ball milling on the structural, thermal, and rheological properties of oat bran protein flour. They observed modifications in the structural conformation of both protein and starch as a consequence of the reduction of the particle size and the suppression of the amylose–lipid complex helical structure. The authors of [17] proved that the cellulose crystallite size decreased as a function of increasing milling time.

It has been recognized that the blending of hydrocolloids and cereal starches of various origin, apart from the main above-mentioned standard applications, also plays a critical role in improving both the structure and texture of yeast-leavened baked products for celiac patients. In such products, in which starch is the main component, two of the most used ingredients, due to their wide availability, low price, and desirable rheological properties, are corn and rice (both flour and starch) [7]. In this context, with the aim to achieve suitable functional properties in the development of optimized gluten-free products, it was decided to use rice flour and corn starch as basic ingredients of the starch–flour system.

Starting from these points, the objectives of this study were to explore the interactions of three different hydrocolloids, namely, guar gum, tara gum, and methylcellulose, with starch components when they are used alone or in blends in a corn starch–rice flour system and to analyze the effect of the ball milling treatment on the structure and viscosity of these different hydrocolloids. With this aim, X-ray diffraction (XRD) experiments were carried out to evaluate the potential changes in the conformational structure induced by the ball milling treatment, while the gelling capacity test was conducted to determine potential changes in the viscosity of hydrocolloid aqueous solutions. Viscometric and small deformation rheological measurements were conducted in binary and ternary starch–hydrocolloid mixtures and were compared with the basic starch–flour system (used as a reference sample) to study the potential changes in hydrocolloid functionality in such a complex system.

## 2. Materials and Methods

### 2.1. Raw Materials

Rice flour, corn starch, and guar gum (GG) were provided by Chimab (Chimab Food Ingredient Solutions, Campodarsego, PD, Italy); tara gum (TG), under its commercial name AGLUMIX 01, was obtained from Silvateam Food Ingredients (Silvateam Food Ingredients S.r.l., San Michele Mondovì, CN, Italy); methylcellulose (MC) was purchased from Sigma–Aldrich.

### 2.2. Ball Milling Treatment

Ball milling was performed using a Mixer/Mill 8000 (SPEX SamplePrep, Metuchen, NJ, USA) with a zirconia jar and two zirconia balls (2 g each); 15 g of hydrocolloids was introduced in the vial and milled for 1 h at 875 rpm under air.

### 2.3. X-ray Diffraction Experiments

The X-ray diffraction patterns of both milled and un-milled hydrocolloids were collected using a SmartLab X-ray powder diffractometer (Rigaku, Tokyo, Japan) aligned according to Bragg–Brentano geometry with Cu Kα radiation (λ = 1.54178 Å) and a graphite monochromator in the diffracted beam. Since the polymers showed broad haloes typical of an amorphous condition, the reciprocal space investigation was deliberately restricted in the angular range from 5° to 60° in 2θ, which allows us to determine the main shape features of patterns.

### 2.4. Fourier Transform Infrared Analysis

Fourier transform infrared (FTIR) analysis was performed on the examined hydrocolloids, both before and after milling, using an infrared Vertex 70 interferometer (Bruker, Billerica, MA, USA). All samples were ground with ultra-dry KBr (potassium bromide), at a ratio of 1:100, and then pressed into the shape of pellets by applying a uniaxial pressure using a hydraulic press. The spectra were recorded on the hydrogels after drying at 80 °C for 24 h, in transmission mode, in the 400–4000 cm^−1^ range by averaging 64 scans with 4 cm^−1^ resolution. The background was evaluated by measuring the signals of dried air.

### 2.5. Gelling Capacity of Hydrocolloids

The gelling properties of both milled and un-milled hydrocolloids were determined by a Rapid Visco Analyzer (RVA-4, Newport Scientific, Warriewood, NSW, Australia) using the official method RVA 41.02. Before staring the test, both guar and tara gums (0.28 g) were thoroughly dispersed in 28 mL of distilled water at room temperature, while the methylcellulose aqueous solution was prepared by dispersing the powder in hot water (80 °C) until complete solubilization was reached. The cooling profile used to describe the gelling/thickening behavior of the obtained hydrocolloid aqueous solutions was as follows: samples were held at 80 °C for 5 min, gradually cooled to 20 °C at a rate of 1 °C/min, and finally kept at 20 °C for 5 min. All experiments were performed in duplicate.

### 2.6. Starch/Flour–Hydrocolloid Blend Preparation

To verify changes in the hydrocolloid functionality induced by the ball milling treatment, both milled and un-milled hydrocolloids were added to a starch-based system composed of corn starch and rice flour (50:50). To this end, twelve different hydrocolloid–starch combinations, which included six binaries and six ternary mixtures, were obtained (Table 1). Firstly, each milled and un-milled hydrocolloid was individually added to the basic starch–flour system to obtain six binary mixtures; secondly, blends of two hydrocolloids, which were obtained by combining the gelling agent methylcellulose (both milled and un-milled) with each of the two thickening agents (both milled and un-milled), were added to the basic starch–flour system to obtain six ternary mixtures. The corn starch–rice flour system alone was used as a reference sample (Table 1).

To maintain a constant concentration of starch in both reference and experimental samples, thus allowing comparison among them, the starch–hydrocolloid composites were obtained by addition. The blending of a single hydrocolloid and starch–flour was prepared by firstly suspending the hydrocolloid powder (0.25 g) in 25 mL of distilled water at room temperature and under magnetic stirring, before adding 3.5 g of corn starch–rice flour (50:50). The ternary mixtures were prepared in the same way but, in this case, both hydrocolloids (0.125 g + 0.125 g) were simultaneously suspended in the distilled water before adding the starch powder (Table 1). On the contrary, for all the mixtures containing MC, the hydrocolloid solutions were prepared by dispersing the powder in hot water (80 °C) until a complete solubilization of the particles was achieved; then, the obtained hydrocolloid aqueous solutions were cooled to room temperature and subsequently mixed with the corn starch–rice flour powder. Two different batches of each mixture were prepared, and all the measurements were performed in duplicate.

### 2.7. Pasting Properties of Starch/Flour–Hydrocolloid Blends

Viscosity profiles of the starch–flour system alone and the starch/flour-hydrocolloid aqueous solutions were determined by a Rapid Visco Analyzer (RVA-4, Newport Scientific, Warriewood, NSW, Australia) according to the International Association of Cereal Science and Technology (ICC) Standard method 162. Before starting the test, the slurries were heated at 50°C and stirred for 5 min at a rotation speed of 960 rpm to achieve complete homogenization. Then, the slurries were held at 50 °C for 1 min, gradually heated to 95 °C, held at this temperature for 2 min 30 s, and finally cooled to 50 °C and held at this temperature for 2 min. The pasting temperature (°C), peak time (when peak viscosity occurred; min), peak viscosity (maximum hot paste viscosity), breakdown (peak viscosity minus holding strength or minimum hot paste viscosity), setback (final viscosity minus holding strength or minimum hot paste viscosity), and final viscosity (end of the test after cooling to 50 °C and holding at this temperature) were determined. All the viscosity parameters are expressed in mPa⋅s.

### 2.8. Viscoelastic Properties of Starch/Flour–Hydrocolloid Blends

Immediately after pasting, the obtained starch–flour and starch/flour–hydrocolloid gels were subjected to small deformation rheological measurements using an MCR 92 rotational rheometer (Anton Paar GmbH, Inc., Graz, Austria) equipped with a Peltier-temperature-controlled system, as previously suggested by [18]. The test was performed at 50 °C using a 60 mm serrated plate–plate geometry with a 2 mm gap between plates. After lowering the upper plate, the excess sample was trimmed off and a thin layer of silicon oil was used to cover the exposed surface to prevent moisture losses during the test. Prior to starting the test, the experimental gels were rested for 5 min between plates to allow sample relaxation. The frequency sweep test was carried out over the range 0.1–10 Hz at 50 °C using a target strain of 0.01%, which fell within the linear viscoelastic region previously determined by running a strain sweep test at a constant frequency of 10 Hz and with a strain that varied over the range 0.001–100. Values of storage modulus (G′), loss modulus (G″), and loss tangent (tan *δ*) were recorded at a frequency of 1 Hz.

### 2.9. Statistical Analysis

The experimental data obtained from viscometric and rheological measurements were analyzed using one-way analysis of variance (ANOVA). Fisher’s least significant differences (LSD) test was applied to determine the difference between each pair of means with 95% confidence (*p* < 0.05). Statistical analysis of the results was performed using Statistica v10.0 software (StatSoft, Inc., Tulsa, OK, USA).

## 3. Results and Discussion

### 3.1. Structural and Viscosity Properties of the Single Hydrocolloids

**Figure 1:**
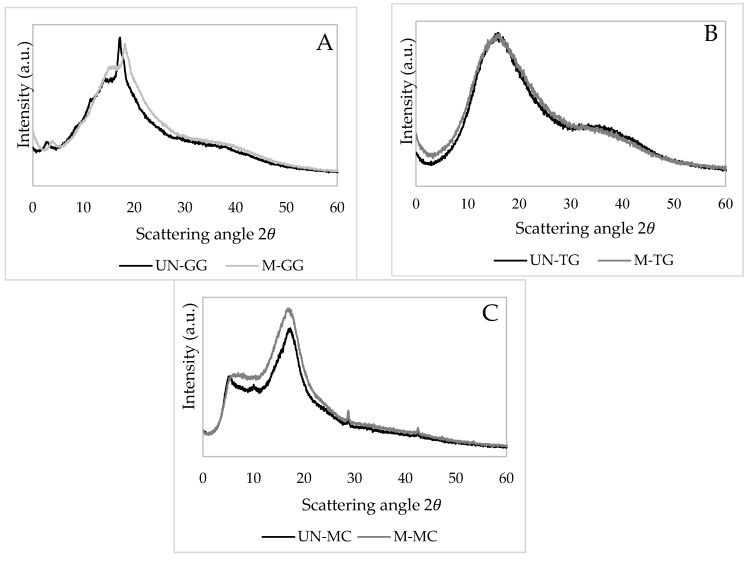
X-ray diffraction patterns of un-milled (continuous dark line) and milled (continuous grey line) hydrocolloids: guar gum (**A**), tara gum (**B**), and methylcellulose (**C**). UN: un-milled; M: milled; GG: guar gum; TG: tara gum; MC: methylcellulose.

Guar, tara, and methylcellulose powders were analyzed by a wide-angle X-ray diffraction technique in order to evaluate the effect of the mechanical treatment on the structural evolution of the three hydrocolloids (Figure 1).

The XRD patterns of the un-milled GG (UN-GG) and milled GG (M-GG) powders are presented in Figure 1A. Both guar systems exhibited a typical XRD profile with very low crystallinity (2θ = 17.02 and 20.11°) with respect to the amorphous counterpart (broad peak centered at 18.1° in 2θ). After the ball milling, a pronounced reduction in crystallinity (see peak at 17.02°), which could be ascribable to the partial hydrogen bonds breaking the guar structure, is observed [19,20].

Figure 1B depicts the XRD pattern of the TG before and after milling. The two broad peaks centered at 18.6 and 39.8° in 2θ are typical of an amorphous pattern. The effect of milling on these powders was not comparable with the previous system: the analysis of the peak shape and broadening revealed an increase of the amorphous component of 2%–4% for the milled sample. For this reason, a clear effect of the mechanical treatment cannot be shown by X-ray diffraction for the TG sample. FT-IR analysis, not shown here, seemed to confirm the experimental evidence achieved by X-ray diffraction.

XDR patterns for MC samples are shown in Figure 1C. The observed diffraction peaks for both materials can be attributed to the crystalline scattering and the diffuse background of the disordered regions. The diffractogram corresponding to the un-milled MC (UN-MC) sample showed maximum diffraction peaks at the 8.1° and 20.2° 2θ angles, while 8.1° and 19.7° 2θ angles were recorded for the ball milled MC (M-MC). Comparing the XRD patterns, it is possible to observe a shift versus a lower 2θ angle of the main peak for the ball-milled system. This indicates an increase of the inter-planar distance compared to the original MC, due to the generation of disorder when the MC is ball milled. The maximum, around 20°, present in the MC sample, is commonly known as van der Waals halo, which appears for many polymers and corresponds to the polymeric chain packing due to van der Waals forces [21]. Also, the maximum around 8°, which is known as the halo of low van der Waals, occurs for some amorphous polymers due to the existence of regions with aggregates of segments of parallel chains. The peak broadening (b–violet curve) is a direct consequence of the mechanical treatment which induces a further decrease of the crystallinity of the MC powders (35%). In the case of the native MC, this maximum is much more defined, conferring this sample a more semicrystalline character (46%).

The gelling behavior of the investigated hydrocolloids, both before and after milling, were then analyzed as a function of the time to determine potential changes in the viscosity properties of the hydrocolloid aqueous solutions.

**Figure 2:**
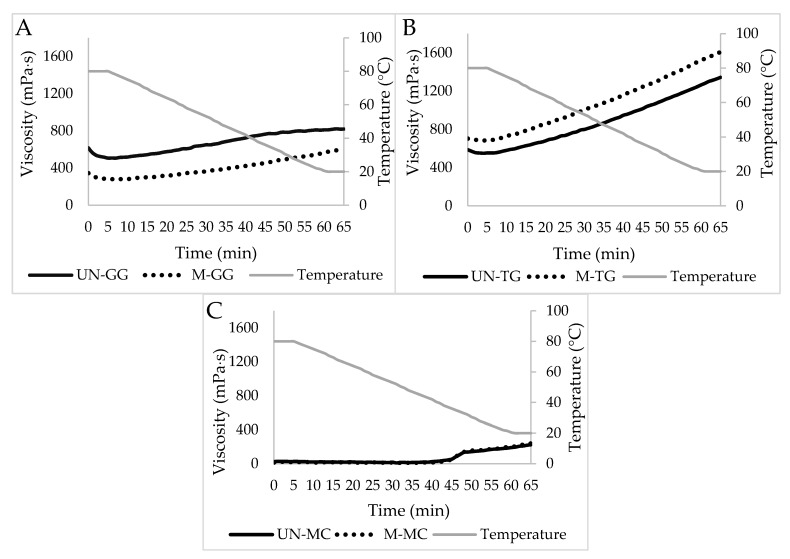
Gelling/thickening profiles of the three un-milled (continuous line) and milled (discontinuous line) hydrocolloids: guar gum (**A**), tara gum (**B**), and methylcellulose (**C**). UN: un-milled; M: milled; GG: guar gum; TG: tara gum; MC: methylcellulose.

The difference in viscosity due to the ball milling treatment is clearly visible in Figure 2. In this case, the effect of ball milling on the guar gum resulted in a diminished viscosity due to the fact that the crystalline domains decreased in length and therefore the chains flowed better (Figure 2A). On the contrary, the greater affinity due to the overexposure of OH groups of the hydrocolloid chains turned out to be the dominant effect in tara gum aqueous solutions, which showed increased values of viscosity (Figure 2B).

Methylcellulose appeared to have an intermediate behavior between the two effects, as if they were subjected to compensation. In fact, there was no substantial difference between the UN-MC and the M-MC. The RVA analysis graph (Figure 2C) showed an important characteristic of methylcellulose: its lower critical solution temperature (LCST). LCST is a change in the MC microstructure, passing from a hydrophilic to a hydrophobic state as a consequence of a change of temperature [22]. The behavior of this hydrocolloid is not monotonous when the temperature varies; the viscosity of an MC solution slightly decreases below a critical temperature value over which the viscosity regime increases [23]. In the RVA graph, the change in viscosity was between 40 and 50 °C.

### 3.2. Properties of Binary Hydrocolloid–Starch Blends

The viscometric properties of a corn starch-rice flour system in the absence and presence of milled and un-milled hydrocolloids are reported in Table 2. The effects of both pure hydrocolloids and the ball milling treatment are explored first, prior to analyzing the influence of different combinations of pairs of hydrocolloids on the pasting profiles of the resulting starch–hydrocolloid blends.

**Figure 3:**
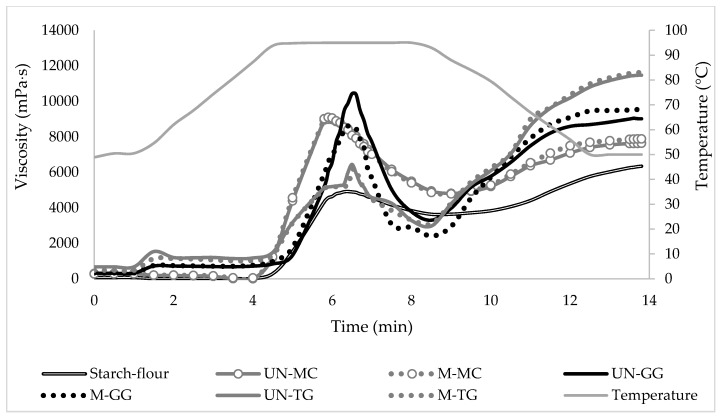
Viscometric profiles of the corn starch/rice flour system in the absence and presence of single milled and un-milled hydrocolloids. UN: un-milled; M: milled; GG: guar gum; TG: tara gum; MC: methylcellulose.

Among the tested samples, the starch–flour system alone exhibited lower values of peak viscosity, breakdown, setback, and final viscosity than those observed when the single hydrocolloids were added to the starch paste. Only the MC, irrespective of whether it had been treated or not, showed values of setback and final viscosity during the gelling/cooling stage similar to those of the individual starch (Table 2). A possible explanation for such an increase in viscosity could be that starch–hydrocolloid dispersions are complex biphasic systems composed of a suspension of swollen granules dispersed in a continuous medium in which amylose, low-molecular-weight amylopectin molecules and the added hydrocolloids are located [24,25]. As gelatinization and pasting proceed, the starch granules continue to swell and, in turn, to absorb and bind more water, leading to a reduction of the volume occupied by the hydrocolloids that, being more concentrated, cause a rise in the viscosity of the entire system [24]. Furthermore, the thickening ability of hydrocolloids as well as interactions between hydrocolloids and swollen starch granules and/or leached amylose may also be involved as other concurrent factors [26]. However, while the addition of both milled and un-milled hydrocolloids was significant in promoting the viscosity levels, each hydrocolloid affected the pasting properties of the starch system in a different way (Figure 3). In fact, during gelatinization and pasting, the viscosity profiles of the starch/GG mixtures were higher than those shown by the other galactomannan TG regarding peak viscosity and breakdown, whereas intermediate viscosity values were observed with the addition of the MC, in which the values of peak viscosity were significantly higher than those of the TG mixtures, but the values of breakdown were significantly lower than those of guar gum blends. Discussing the results more in depth, it can be observed that within each tested hydrocolloid, the effect of the ball milling treatment was found to be significant only in the case of GG, in which the mechanochemical treatment seemed to have some detrimental effect on peak viscosity (Table 2). However, the UN-GG was the hydrocolloid that most strongly affected both peak viscosity and breakdown of the starch–flour mixture. This result could be explained by assuming that, during pasting, this hydrocolloid is not able to cover or interact with starch granules, but persists in the macromolecular medium which forms a sheet structure [27]; in this way, it does not hinder the swelling of the starch granules, which are free to swell, leading to a great increase in peak viscosity. After that, the more fragile swollen granules, becoming less resistant to the mechanical shearing, lose their integrity, leading to a severe drop (the so-called breakdown) in the viscosity of the system that becomes, in turn, less stable [25]. The M-GG, on the contrary, when added to the corn starch-rice flour mixture behaved in a different way, resulting in a lower and flatter peak viscosity. To better understand this different behavior, it must be borne in mind that, due to the reduction of the crystalline domains caused by the ball milling treatment, the M-GG exhibited more fluidity than the UN-GG. Therefore, it can be hypothesized that this decrease in peak viscosity (in line with the results obtained in the gelling capacity analysis) may be due to the smooth flow that the M-GG molecules exhibit in the continuous phase without interacting with the leached amylose and amylopectin [28]. Similar conclusions were reported by [28] to explain the decrease in the peak viscosity observed when low-molecular-weight GG was added to a corn starch–rice flour system.

As in the case of the GG, the TG, irrespective of whether it had been treated or not, also increased the peak viscosity, but the extent of this increase was significantly lower than that observed for GG (Table 2). Furthermore, while the TG showed a lower breaking viscosity during heating, it promoted higher stability of the system. The authors of [26], when studying the interactions between wheat starch and both guar and tara gums, reported opposite results to our findings. In fact, these authors observed higher values of peak viscosity using the TG, whereas they found similar values of breakdown and setback in the TG-containing samples compared to the guar gum mixtures. This contrasting behavior, however, may be due to the different type of starch and/or to the different method of sample preparation used. Furthermore, in contrast to what was previously reported for the gelling capacity of the milled TG (M-TG) dispersed in an aqueous environment, the ball milling treatment seemed to have no effect on the pasting properties of the TG-starch blend. Presumably, in a complex system such as starch–hydrocolloid mixtures, the overexposure of the hydroxyl groups previously hypothesized for the TG as a consequence of the ball milling treatment, was counterbalanced by the competition for water with the starch granules.

When hot pastes are cooled to 50 °C, a transition from a viscous liquid to a gel, the so-called setback, takes place, leading to an increase in the viscosity of the system primarily due to the re-association of amylose molecules [29]. During this stage, the values of setback and final viscosity were found to be higher than those of the individual starch system for both GG and TG, suggesting that both galactomannans may have a marked tendency to promote starch retrogradation (Table 2). In the case of the UN-GG, however, the extent of such an increase was significantly lower (Figure 3). The differences in the molecular structure of these two galactomannans, especially in terms of the mannose/galactose ratio (3:1 for TG and 2:1 for GG) [10], could explain the differences in the gelling properties of the resulting starch–hydrocolloids blends. In fact, as reported by [30], the presence of a higher number of galactose side chains, which impart molecular flexibility, may facilitate intermolecular associations between guar gum and starch components, delaying, in turn, starch retrogradation of the resulting blends. Moreover, an increase in the effective concentration of amylose in the continuous phase, which leads to faster amylose gelation, could be considered as one of the main factors involved in promoting starch retrogradation [25,26].

Unlike the galactomannans, the addition of the cellulose derivative MC, irrespective of whether it had been treated or not, affected the pasting properties of the starch–flour system only during the heating stage (Figure 3). In fact, values of setback and final viscosity were found to be similar to those of the starch–flour system alone. As previously seen for both the GG and TG, the MC also increased the peak viscosity but, in this case, the amount of time to reach the peak was shorter (Table 2).

However, considering that the MC has the unique property needed to forming gels at temperatures above 60 °C [9], this early increase in the peak viscosity might be related to the hydrocolloid gelation rather than to an effective ability of the MC to assist granule swelling during heating. On the contrary, as previously observed by [31] in potato starch-MC composites, the ability of the MC to prevent starch retrogradation could be explained due to its water solubility. Presumably, the MC, acting as a water binder, could cause either the reduction of the available water required by amylose for the recrystallization process to occur or the inhibition of the possible cross-links between the hydrocolloid and the amylose molecules.

In summary, it can be said that both a cellulose derivative and galactomannans modified the gelatinization and retrogradation behavior of the corn starch–rice flour system in ways that vary depending on their different molecular structure and functionality. On the contrary, the conformational changes induced by mechanochemical treatment have not proven to be effective in changing the behavior of the tested hydrocolloids, except in the case of guar gum.

### 3.3. Pasting Properties of Ternary Hydrocolloid–Hydrocolloid–Starch Blends

Considering that, in many food systems, the inclusion of a single hydrocolloid might be not sufficient to develop final products with specific attributes in terms of structure, viscosity, stability, and functionality, the effects of combinations of two hydrocolloids on the viscometric properties of a corn starch–rice flour system were also analyzed. It is common agreement that the blending of two hydrocolloids with different functionality in food systems, such as gelling and thickening agents, can have synergistic effects, conferring enhanced viscosity and improved or induced gelation [32,33]. In this regard, six different combinations of both treated and un-treated hydrocolloids were studied: firstly, the UN-MC was combined with each of the two tested galactomannans either before and after the ball milling treatment and, secondly, the M-MC was combined with each of the milled galactomannans.

**Figure 4:**
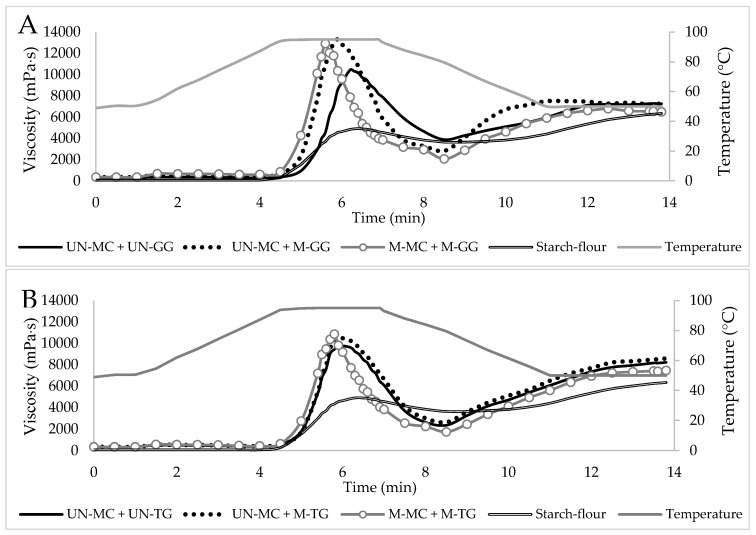
Viscometric profiles of the corn starch-rice flour system in the absence and presence of two milled and un-milled hydrocolloids. MC-GG-starch/flour (**A**) and MC-TG-starch/flour (**B**) ternary mixtures. UN: un-milled; M: milled; GG: guar gum; TG: tara gum; MC: methylcellulose.

As reported in Figure 4, the addition of two hydrocolloids, instead of one, significantly changed the viscometric profile of the starch–flour system during both heating and cooling cycles, not only when compared to the individual starch, but also in comparison with the binary blends (Table 2). Among the tested samples, no significant differences were observed only in the pasting temperature parameter, whereas, as stated above, the decreased times to reach the peak viscosity observed in all the experimental blends prepared with the addition of MC were probably due to the thermogelation properties of this cellulose derivative.

Analyzing the results more in depth, it can be observed that the highest peak viscosity and breakdown were obtained in those blends in which the milled guar gum was combined with both the M- and UN-MC (Table 2). These results, being even higher than those that occurred in each hydrocolloid individually, suggested additive interactions between these two gums. A possible explanation for such an increase in viscosity could be that the MC, acting as a gelling agent, is able to build up a network in which the GG, acting as thickening agent, fills up the spaces [32]; in this way, the concentration of the continuous phase increases, causing, in turn, a rise in viscosity of the entire system. Moreover, considering that such an increase was less pronounced when the two hydrocolloids were combined in their native form, it can be hypothesized that the structural changes induced by the ball milling treatments (especially for guar gum) may have facilitated such heterotypic interactions. In addition, during the cooling stage, the values of setback and final viscosity, being lower than those observed for the M-GG/starch mixture, suggested that the addition of both the M- and UN-MC may promote a marked tendency to prevent starch retrogradation of the resulting ternary blends.

As in the case of the M-GG, the TG used in combination with both the M- and UN-MC also increased peak viscosity and breakdown of the starch–flour system, but the effect was less pronounced when the two hydrocolloids were combined in their native form (Figure 4B). During the cooling stage, however, lower values of final viscosity and setback were observed as compared to the binary mixture with added the M-TG, but higher values of setback and similar values of final viscosity were obtained as compared to the binary mixture with both the M- and UN-MC (Table 2). On the basis of these results, it can be hypothesized that, although there have been additive interactions between the two hydrocolloids, the blending of MC and GG seemed to be more effective in modifying the viscometric profile of the corn starch–rice flour system.

### 3.4. Viscoelastic Properties of Hydrocolloids Starch Gels

The viscoelastic parameters of the corn starch/rice flour system in the absence and presence of milled and un-milled hydrocolloids were measured at 50 °C immediately after pasting.

In all the tested samples, the storage modulus (G′), the elastic component of the material, was higher than the loss modulus (G″), the viscous part of the material, indicating that all the starch pastes had a solid, elastic-like behavior typical of a biopolymer gel (Table 1). However, for all the tested mixtures, values of both dynamic moduli did not differ significantly from those of the starch alone, except for the UN-GG/starch and M-TG/starch gels, in the case of the binary mixtures, and the M-MC/M-TG/starch gels, in the case of the ternary mixtures. Such increases in the viscoelastic properties observed in both mixtures prepared with the M-TG, as well as in the binary mixture prepared with the UN-GG, may be attributed to an increase in the viscoelastic properties of the individual gums. However, the mechanochemical treatment seemed to affect the galactomannans behavior in opposite ways. In fact, in the case of GG, the elastic properties of the starch-hydrocolloid system were found to be higher than those of the individual starch only when the gum was added in its pure form, suggesting that the mechanochemical treatment might have lowered the tendency of the GG to form gels, probably as a result of increased synergistic interactions. On the contrary, in the blends obtained by adding the UN-TG, considering that no differences in the values of both dynamic moduli (apart from a slight increase in the binary mixture) were observed in comparison to the individual starch (Table 3), it can be assumed that the ball milling treatment may have favorably affected the hydrocolloid behavior in this complex system.

Moreover, analyzing the results more in depth, it can be observed that the addition of the M-TG into the starch–flour system led to an increase in G″ values more pronounced than that observed in G’, indicating that this galactomannan was more effective in increasing the viscous properties of the system rather than the elastic properties (Table 3). A possible explanation for these results could be related to the phase separation between starch components and galactomannan that takes place in the dispersed phase as a result of thermodynamic incompatibility between these two chemically dissimilar polymers [24]. In this way, intramolecular interactions may be energetically promoted in comparison with intermolecular associations, modifying the viscoelastic properties of the resulting starch–hydrocolloid gels. Similar conclusions were reported by [34] to explain the increase in the viscous properties of a rice starch–flour system when increasing concentration levels of the TG (0.2%–0.6%) are added to it. In addition, although no significant differences were observed in the tan *δ* values of all the hydrocolloid-starch pastes with respect to the individual starch, the highest values of the ratio G″/G′ shown by both the M-TG starch mixtures seemed to further confirm the results described above.

## 4. Conclusions

On the basis of the data obtained by XRD experiments and gelling tests, it could be said that the ball milling treatment affected the structure of the tested hydrocolloids and, in turn, the viscosity of their aqueous solutions in different ways. On the one hand, in the case of the GG, the reduction of the crystalline domains and the consequent increase in the fluidity of the system induced by the mechanochemical treatment seemed to be the main causes of the decrease in viscosity observed when the M-GG aqueous solutions were compared with those prepared with the UN-GG; on the other hand, in the case of the TG, the larger exposure of the hydroxyl groups seemed to justify the increase in viscosity observed in the M-TG aqueous solutions when compared to those prepared with the UN-TG, even if it was not possible to confirm this effect with XRD and IR analysis. On the contrary, the MC seemed to have an intermediate behavior between the two effects, leading to aqueous solutions with the same viscosity.

When each hydrocolloid was individually combined with a starch–flour system, these same trends were observed in the viscometric profiles of the starch–hydrocolloid systems, with the exception of starch-TG blends in which no differences between blends containing the M- and the UN-forms were observed. However, considering that the conformational changes induced by the mechanochemical treatment seemed to be effective only in the case of the GG, it could be assumed that the different pasting properties observed in the binary starch–hydrocolloid mixtures in comparison to the starch alone might be due to the type of hydrocolloid added rather than to structural changes induced by the treatment. On the contrary, the ball milling treatment seemed to be more effective when two combined hydrocolloids, especially when they were both in the milled form, were simultaneously added to the starch–flour system. On the contrary, the dynamic moduli of the cornstarch–rice flour gels were significantly affected by the addition of the hydrocolloids only in the case of the UM-GG/starch, M-TG/starch, and M-MC/M-TG/starch blends, indicating that the ball milling treatment was involved in promoting phase separation between starch and M-TG molecules in the dispersed phase to a greater extent in comparison to the guar systems.

This study represents a preliminary attempt to understand how the effects induced by the ball milling treatment on different types of hydrocolloids may change their functionality not only when they are used alone, but also when they are included in a complex system, such as a starch–flour system. Further experiments are needed to analyze more in depth which intra- and inter-molecular interactions, occurring between starch and treated hydrocolloids and within the same hydrocolloid molecules, may be involved in changing the paste and gel behavior of starch–hydrocolloid systems.

## Figures and Tables

**Table 1:** Formulations used to prepare the starch/flour-hydrocolloid blends.

Samples	Corn Starch (g)	Rice Flour (g)	Guar Gum (g)		Tara Gum (g)		Methylcellulose (g)		Water (mL)
			UN	M	UN	M	UN	M	
Corn starch–rice flour	1.75	1.75	-	-	-	-	-	-	25
*Binary mixtures*									
starch–flour + UN-GG	1.75	1.75	0.25	-	-	-	-	-	25
starch–flour +M-GG	1.75	1.75	-	0.25	-	-	-	-	25
starch–flour + UN-TG	1.75	1.75	-	-	0.25	-	-	-	25
starch–flour + M-TG	1.75	1.75	-	-	-	0.25	-	-	25
starch–flour + UN-MC	1.75	1.75	-	-	-	-	0.25	-	25
starch–flour + M-MC	1.75	1.75	-	-	-	-	-	0.25	25
*Ternary mixtures*									
starch–flour + UN-MC + UN-GG	1.75	1.75	0.125	-	-	-	0.125	-	25
starch–flour + UN-MC + M-GG	1.75	1.75	-	0.125	-	-	0.125	-	25
starch–flour + M-MC + M-GG	1.75	1.75	-	0.125	-	-	-	0.125	25
starch–flour + UN-MC + UN-TG	1.75	1.75	-	-	0.125	-	0.125	-	25
starch–flour + UN-MC + M-TG	1.75	1.75	-	-	-	0.125	0.125	-	25
starch–flour + M-MC + M-TG	1.75	1.75	-	-	-	0.125	-	0.125	25

UN: un-milled; M: milled; GG: guar gum; TG: tara gum; MC: methylcellulose.

**Table 2:** Viscometric parameters of corn starch–rice flour systems formulated with one or two un-milled and milled hydrocolloids.

Samples	Parameters																							
	Peak Viscosity (mPa⋅s)				Breakdown (mPa⋅s)				Final Viscosity (mPa⋅s)				Setback (mPa⋅s)				Peak Time min				Pasting Temperature °C			
**Corn starch–rice flour**	**4917**	**±**	**192**	**a**	**1300**	**±**	**54**	**a**	**6360**	**±**	**328**	**a**	**2743**	**±**	**82**	**a**	**10.53**	**±**	**0.0**	**ef**	**77.08**	**±**	**0.60**	**ns**
***Single Hydrocolloid + Starch–flour***																								
UN-GG	10431	±	430	ef	7168	±	130	cde	9026	±	86	b	5763	±	385	bc	10.70	±	0.0	f	78.23	±	2.30	ns
M-GG	8681	±	730	c	6337	±	1080	c	9529	±	645	bc	7185	±	296	cd	10.57	±	0.0	f	76.30	±	2.90	ns
UN-TG	6732	±	730	b	3854	±	203	b	11,493	±	1520	c	8615	±	1201	d	10.60	±	0.0	f	76.68	±	0.00	ns
M-TG	6436	±	613	b	3508	±	185	b	11,651	±	1641	c	8723	±	1213	d	10.70	±	0.0	f	76.60	±	1.00	ns
UN-MC	9031	±	716	cd	4224	±	938	b	7644	±	5	ab	2837	±	216	a	10.00	±	0.2	abc	76.33	±	0.60	ns
M-MC	9115	±	270	cd	4362	±	412	b	7912	±	186	ab	3158	±	328	a	10.07	±	0.1	c	76.65	±	1.20	ns
***Pairs of Hydrocolloids + Starch–flour***																								
UN-MC + UN-GG	10,428	±	107	ef	6586	±	35	cd	7299	±	370	ab	3457	±	228	a	10.33	±	0.0	de	78.20	±	1.20	ns
UN-MC + M-GG	13,336	±	434	g	10,527	±	533	g	7277	±	231	ab	4468	±	132	ab	10.04	±	0.0	bc	78.58	±	0.60	ns
M-MC + M-GG	12,980	±	129	g	10,934	±	18	g	6480	±	2948	a	4434	±	2801	ab	9.80	±	0.2	a	78.60	±	0.50	ns
UN-MC + UN-TG	9778	±	454	de	7526	±	260	de	8246	±	204	ab	5994	±	9	bc	10.17	±	0.0	cd	78.70	±	1.80	ns
UN-MC + M-TG	10,573	±	134	ef	7981	±	362	e	8619	±	88	ab	6028	±	408	bc	10.10	±	0.0	c	79.45	±	0.60	ns
M-MC + M-TG	10,948	±	202	f	9235	±	418	f	7445	±	148	ab	5731	±	68	bc	9.84	±	0.2	ab	77.83	±	1.60	ns

Mean values ± standard deviation. Within columns, values (mean of two replicates) followed by the same letter do not differ significantly from each other (*p* ≤ 0.05). UN: un-milled; M: milled; GG: guar gum; TG: tara gum; MC: methylcellulose.

**Table 3:** Viscoelastic properties of corn starch–rice flour systems formulated with one or two un-milled and milled hydrocolloids.

Samples	Storage Modulus G′ (Pa)				Loss Modulus G″ (Pa)				Loss Tangent *tan δ*			
**Corn starch–rice flour**	**1449**	**±**	**191**	**abc**	**147**	**±**	**0**	**a**	**0.103**	**±**	**0.013**	**ns**
***Single Hydrocolloid + Starch–flour***												
UN-GG	2024	±	320	d	234	±	12	ab	0.117	±	0.012	ns
M-GG	1222	±	227	a	151	±	31	a	0.124	±	0.002	ns
UN-TG	1785	±	105	cd	315	±	66	ab	0.176	±	0.026	ns
M-TG	2887	±	173	e	635	±	335	c	0.217	±	0.103	ns
UN-MC	1254	±	161	ab	212	±	20	ab	0.170	±	0.005	ns
M-MC	1287	±	230	ab	239	±	66	ab	0.185	±	0.018	ns
***Pairs of Hydrocolloids + Starch–flour***												
UN-MC + UN-GG	1594	±	101	abcd	245	±	50	ab	0.153	±	0.021	ns
UN-MC + M-GG	1705	±	201	bcd	252	±	3	ab	0.149	±	0.016	ns
M-MC + M-GG	1816	±	34	cd	284	±	37	ab	0.157	±	0.018	ns
UN-MC + UN-TG	1379	±	14	abc	237	±	6	ab	0.172	±	0.002	ns
UN-MC + M-TG	1751	±	523	cd	312	±	113	ab	0.177	±	0.012	ns
M-MC + M-TG	1942	±	26	d	399	±	110	b	0.206	±	0.059	ns

Mean values ± standard deviation. Within columns, values (mean of two replicates) followed by the same letter do not differ significantly from each other (*p* ≤ 0.05). UN: un-milled; M: milled; GG: guar gum; TG: tara gum; MC: methylcellulose.
